# Supplementary material for: Profiling protein–protein interactions to predict the efficacy of B-cell-lymphoma-2-homology-3 mimetics for acute myeloid leukaemia
Source: Nat Biomed Eng. 2024 Jul 18;8(11):1379–95. doi: 10.1038/s41551-024-01241-3 (PMC11584402; doi:10.1038/s41551-024-01241-3)
Supplement: Supplementary file 1 — Supplementary tables. [file 41551_2024_1241_MOESM1_ESM.pdf]

nature biomedical engineering

Supplementary information https://doi.org/10.1038/s41551-024-01241-3

Profiling protein–protein interactions to predict the efficacy of B-cell-lymphoma-2-homology-3 mimetics for acute myeloid leukaemia

In the format provided by the authors and unedited

Supplementary Table 1 | Comparison of calculated K_d values between each binding pairs regarding binding cooperativity. n is the Hill coefficient fitted from the generalized Hill equation (*Fractional occupancy=([PPI probe]^n/(K_d+[PPI probe]^n)*). The K_d values were ascertained based on the assumption of one-to-one complex formation (fixed n=1) or alternative binding stoichiometries for the binding curves in **Extended Data Fig. 6a-d**.

PBA	Fitted *n*	Fitted K_d (nM)	Fitted K_d (nM) (fixed *n*=1)
BCL2-BIM_{BH3} PBA	0.92 ±0.07	42.3 ± 5.9	49.8 ± 2.2
BCLxL-BIM_{BH3} PBA	1.02 ± 0.09	11.4 ± 2.1	11.0 ± 0.5
MCL1-BIM_{BH3} PBA	0.97 ± 0.08	4.6 ± 0.7	4.6 ± 0.2
BCL2-BIM_{EL} PBA	1.29 ± 0.08	260 ± 47	134 ± 9.4
BCLxL-BIM_{EL} PBA	1.02 ± 0.05	74.0 ± 8.1	71.1 ± 1.9
MCL1-BIM_{EL} PBA	0.99 ± 0.07	57.0 ± 8.0	57.8 ± 2.0
BCL2-BAD PBA	0.96 ± 0.12	5.1 ± 1.0	5.5 ± 0.4
BCLxL-BAD PBA	1.15 ± 0.14	3.2 ± 0.8	2.5 ± 0.2
MCL1-BAD PBA	Not determined	Not determined	Not determined
BCL2-NOXA PBA	Not determined	Not determined	Not determined
BCLxL-NOXA PBA	Not determined	Not determined	Not determined
MCL1-NOXA PBA	0.85 ± 0.24	3.4 ± 1.1	4.1 ± 0.5

v

Supplementary Table 2 | The statistical indicators (coefficients and *p*-values) of each metric for the correlation in Extended Data Fig. 8b (One-sided *F*-test).

Metric	Coefficient	*p*-value
BCL2 total level	0.032	0.32
BCL2-BIM$_{BH3}$ PBA	0.038	0.51
BCL2-BAD PBA	0.041	0.42
BCL2-BAX CPX	0.055	0.15
(intercept)	-1.10	7.00E-06

Supplementary Table 3 | The statistical indicators (coefficients and *p*-values) of each metric for the correlation in Fig. 4f (One-sided *F*-test).

Metric	Coefficient	*p*-value
BCL2-BIM_{BH3} PBA	0.097	0.01
BCL2-BAX CPX	0.11	0.02
BCLxL-BAK CPX	-0.027	0.04
(intercept)	-0.56	0.04

Supplementary Table 4 | Comparison of predictive powers between analysis models (*n*=32). MSE: Mean squared error, AUC: Area under curve, ROC: Receiver operating characteristic.

**Model**	**MSE**	**AUC (from ROC)**
Combination	0.014	0.94
BCL2-BAD PBA	0.016	0.92
BCL2-BIM_{BH3} PBA	0.017	0.88
BCL2-BAX CPX	0.021	0.94
BCL2 total level	0.023	0.90

Supplementary Table 5 | Comparison of the predictive powers for ABT-199 drug responses across different methods. *n*=14 for BCL2 SMPC and PBA profiling, BH3 profiling, and flow cytometry, and *n*=32 for western blotting.

**Method**	**Pearson R**	**MSE**	**AUC (from ROC)**
BCL2 SMPC and PBA profiling	0.86	0.009	0.93
BH3 profiling	0.65	0.019	0.80
Flow cytometry	0.49	0.025	0.71
Western blotting	0.59	0.036	0.85

Supplementary Table 6 | The statistical indicators (coefficients and *p*-values) of each metric for the correlation in Fig. 5f (One-sided *F*-test).

Metric	Coefficient	*p*-value
MCL1 total level	0.08	1.40E-04
BCLxL-BIM$_{BH3}$ PBA	-0.13	0.003
BCLxL-BAK CPX	-0.05	0.04
(intercept)	2.83	0.003

Supplementary Table 7 | The statistical indicators (coefficients and *p*-values) of each metric for the correlation in Extended Data Fig. 8h (One-sided *F*-test).

Metric	Coefficient	*p*-value
MCL1 total level	0.078	0.02
MCL1-BIM_{BH3} PBA	0.017	0.76
MCL1-NOXA PBA	-0.027	0.67
MCL1-BAK CPX	-0.057	0.30
(intercept)	0.28	0.37

Supplementary Table 8 | Evaluation of the BCL2 SMPC and PBA profiling in predicting *in vivo* clinical response for ABT-199 (*n*=10). The estimated scores were calculated from the model in Fig. 4e. R: Response (CR: Complete remission, CRi: CR with incomplete hematologic recovery, PR: Partial remission), NR: Non-response, *n.a.*: Not available.

Patient No.	Sample No.	Estimated score	*In vivo* response	Note
1	BC-6524	0.90 (R)	R (CRi)	
2	BC-7064	0.78 (R)	R (PR)	+ Azacitidine
3	BC-7082	0.69 (R)	R (PR)	
4	BC-7107-R	0.93 (R)	R (CRi)	+ LDAC
5	BC-7052	0.64 (R)	NR	+ LDAC
6	BC-7081	0.19 (NR)	NR	+ Decitabine
7	BC-7230	0.27 (NR)	NR	
8	BC-8634	0.39 (NR)	NR	+Dacogen
9	BC-8746	0.54 (NR)	NR	+ Azacitidine
10	BC-8784	0.55 (NR)	NR	+Dacogen
	BC-7064-R	0.36	*n.a.*	Relapsed (BC-7064)
	BC-7082-R	0.40	*n.a.*	Relapsed (BC-7082)
	BC-7107-R2	0.20	*n.a.*	Relapsed (BC-7107-R)
	BC-8900	0.47	*n.a.*	After treatment (BC-8634)
	BC-9458	0.59	*n.a.*	After treatment (BC-8746)
	BC-9492	0.60	*n.a.*	After treatment (BC-8784)
