# Supplementary material for: Profiling protein–protein interactions to predict the efficacy of B-cell-lymphoma-2-homology-3 mimetics for acute myeloid leukaemia
Source: Nat Biomed Eng. 2024 Jul 18;8(11):1379–95. doi: 10.1038/s41551-024-01241-3 (PMC11584402; doi:10.1038/s41551-024-01241-3)
Supplement: Supplementary file 2 — Reporting Summary [file 41551_2024_1241_MOESM2_ESM.pdf]

nature portfolio

Corresponding author(s): Tae-Young Yoon
Last updated by author(s): Jan 20, 2024

Reporting Summary

Nature Portfolio wishes to improve the reproducibility of the work that we publish. This form provides structure for consistency and transparency in reporting. For further information on Nature Portfolio policies, see our Editorial Policies and the Editorial Policy Checklist.

Statistics

For all statistical analyses, confirm that the following items are present in the figure legend, table legend, main text, or Methods section.

n/a	Confirmed	
☐	☒	The exact sample size (n) for each experimental group/condition, given as a discrete number and unit of measurement
☐	☒	A statement on whether measurements were taken from distinct samples or whether the same sample was measured repeatedly
☐	☒	The statistical test(s) used AND whether they are one- or two-sided<br>*Only common tests should be described solely by name; describe more complex techniques in the Methods section.*
☒	☐	A description of all covariates tested
☐	☒	A description of any assumptions or corrections, such as tests of normality and adjustment for multiple comparisons
☐	☒	A full description of the statistical parameters including central tendency (e.g. means) or other basic estimates (e.g. regression coefficient) AND variation (e.g. standard deviation) or associated estimates of uncertainty (e.g. confidence intervals)
☐	☒	For null hypothesis testing, the test statistic (e.g. F, t, r) with confidence intervals, effect sizes, degrees of freedom and P value noted<br>*Give P values as exact values whenever suitable.*
☒	☐	For Bayesian analysis, information on the choice of priors and Markov chain Monte Carlo settings
☒	☐	For hierarchical and complex designs, identification of the appropriate level for tests and full reporting of outcomes
☐	☒	Estimates of effect sizes (e.g. Cohen's d, Pearson's r), indicating how they were calculated

Our web collection on statistics for biologists contains articles on many of the points above.

Software and code

Policy information about availability of computer code

Data collection	Single-molecule fluorescence signals were collected using a PI-View (PROTEINA) and assessed using the PI-Analyzer (v1) provided by the manufacturer. The flow-cytometry data were collected using SH800S (Sony) and the SH800S Cell Sorter Software (v2.1.5) provided by the manufacturer. The Western blotting data were collected using ImageQuant LAS 4000 mini (Cytiva) and the ImageQuant TL software (v10.2) provided by the manufacturer.
Data analysis	The PI-Analyzer software (v1) by PROTEINA was used for post-image analysis of the single-molecule fluorescence images. The SH800S Cell Sorter Software (v2.1.5) by Sony was used for flow-cytometry analysis. ImageJ (v1.53a) was used for Western blotting to measure the protein-band intensity. OriginPro (v2022) was used for fitting curves and statistics. Custom graphical user interface (GUI) software written in MATLAB (2021a) and Python (3.11) were used for the analysis of the drug-efficacy prediction model. The custom MATLAB and Python codes are available on GitHub at https://github.com/tyyoonlab-snu/Nat-Biomed-Eng-2023-

For manuscripts utilizing custom algorithms or software that are central to the research but not yet described in published literature, software must be made available to editors and reviewers. We strongly encourage code deposition in a community repository (e.g. GitHub). See the Nature Portfolio guidelines for submitting code & software for further information.

Data

Policy information about availability of data

All manuscripts must include a data availability statement. This statement should provide the following information, where applicable:

- Accession codes, unique identifiers, or web links for publicly available datasets
- A description of any restrictions on data availability
- For clinical datasets or third party data, please ensure that the statement adheres to our policy

The data supporting the findings of this study are available within the article and its Supplementary Information. All raw data generated or analysed during the study are available on GitHub at https://github.com/tyyoonlab-snu/Nat-Biomed-Eng-2023-

Research involving human participants, their data, or biological material

Policy information about studies with human participants or human data. See also policy information about sex, gender (identity/presentation), and sexual orientation and race, ethnicity and racism.

Reporting on sex and gender	All of the patients underwent chromosomal analysis owing to underlying acute myeloid leukemia (AML). Sex was determined on the basis of chromosomal analysis, but sex was not considered in the study design because there is no evidence that ABT-199 responses depend on sex.
Reporting on race, ethnicity, or other socially relevant groupings	35 Korean patients with acute myeloid leukemia undergoing ABT-199 treatment, regardless of previous medical history. All participants were of Asian race and ethnicity, born in Korea. But ethnicity and other socially relevant groupings were not considered in the study design because there is no evidence that ABT-199 responses depend on those groupings.
Population characteristics	35 Korean patients with acute myeloid leukemia undergoing ABT-199 treatment at Seoul National University Hospital were enrolled, regardless of previous medical history. Samples were collected between January 2014 and December 2019. Acute promyelocytic leukemias and biphenotypic leukemias were excluded.
Recruitment	All patients undergoing ABT-199 treatment were asked to participate, and upon agreement their bone-marrow and peripheral-blood samples were collected and their medical history recorded. There were no potential biases.
Ethics oversight	The study was conducted according to the Declaration of Helsinki and was approved by the institutional review board (IRB) at Seoul National University Hospital (IRB number: H-1910-176-107). Informed consent was taken from all patients before participating in any study-related procedure. No compensation was given for their participation.

Note that full information on the approval of the study protocol must also be provided in the manuscript.

Field-specific reporting

Please select the one below that is the best fit for your research. If you are not sure, read the appropriate sections before making your selection.

☒ Life sciences ☐ Behavioural & social sciences ☐ Ecological, evolutionary & environmental sciences

For a reference copy of the document with all sections, see nature.com/documents/nr-reporting-summary-flat.pdf

Life sciences study design

All studies must disclose on these points even when the disclosure is negative.

Sample size	Generally, the single-molecule fluorescence images were obtained from 10 different locations within each chamber of the imaging chip to ensure its statistical repeatability, on the basis of a previous study (Lee, 2013; Yoo, 2016; Lee, 2018). The inter-chip coefficient of variance (CV) of single-molecule co-IP were calculated from 3 independent measurements of protein complexes to ensure statistical repeatability.
Data exclusions	To avoid any biased analysis, the images containing either photo-bleached region or aggregated large dye cluster were excluded after the post-image analysis of PI-View system. The exclusion criteria are described in Extended Data Fig. 2 and in the previous study (Lee, 2013; Yoo, 2016; Lee, 2018).
Replication	Experiments to perform further analysis (including single-molecule fluorescence imaging) were repeated independently at least 2 times, and replications were successful for all experiments. The SMPC for inter-chip CVs were repeated biologically in 3 independent times (Fig. 1 and Extended Data Fig. 3), and replications were successful for all experiments.
Randomization	For all single-molecule fluorescence imaging, each data point was collected from separate wells within the multi-well imaging chips on separate days. The primary AML samples were collected on independent dates and the PPI profiles were measured within the independent days.
Blinding	For the single-molecule fluorescence imaging, blinding was not performed because of the unbiased nature of the in vitro experiments performed in this study.

For ex vivo drug-efficacy analysis, PPI profiling, BH3 profiling and quantitative flow-cytometry analysis of primary AML samples were performed and analysed in a blind manner.

Reporting for specific materials, systems and methods

We require information from authors about some types of materials, experimental systems and methods used in many studies. Here, indicate whether each material, system or method listed is relevant to your study. If you are not sure if a list item applies to your research, read the appropriate section before selecting a response.

Materials & experimental systems

n/a	Involved in the study
☐	☒ Antibodies
☐	☒ Eukaryotic cell lines
☒	☐ Palaeontology and archaeology
☒	☐ Animals and other organisms
☒	☐ Clinical data
☒	☐ Dual use research of concern
☒	☐ Plants

Methods

n/a	Involved in the study
☒	☐ ChIP-seq
☐	☒ Flow cytometry
☒	☐ MRI-based neuroimaging

Antibodies

Antibodies used

Anti-rabbit immunoglobulin G (IgG) with biotin conjugation (111-065-144; Jackson ImmunoResearch, 1:200) and anti-mouse IgG with biotin conjugation (715-066-151; Jackson ImmunoResearch, 1:200) were used to immobilize the antibodies for surface IP. Anti-RFP antibody with biotin conjugation (ab34771; Abcam, 1:200) and anti-GFP antibody with biotin conjugation (ab6658; Abcam, 1:200) were used to immobilize mCherry- or eGFP-labeled proteins on surface. Anti-MCL1 (94296S; Cell Signaling Technology, D2W9E, 1:100), BCLxL (MA5-15142; Thermo Fisher Scientific, C.85.1, 1:100), BCL2 (4223S; Cell Signaling Technology, D55G8, 1:100), BAX (5023S; Cell Signaling Technology, D2E11, 1:100), and BAK (ab32371; Abcam, Y164, 1:100) antibodies were used to immobilize the corresponding proteins via surface pull-down. Anti-MCL1 (MAB8825; Abnova, C9, 1:100), BCLxL (NBP1-47665; Novus Biologicals. OTI4A9, 1:100), BCL2 (sc-7382; Santa Cruz Biotechnology, C2, 1:100), BAX (MABC1176M; Sigma Aldrich, 6A7, 1:100), BAK (sc-517390; Santa Cruz Biotechnology, AT38E2, 1:100), BIM (sc-374358; Santa Cruz Biotechnology, H5, 1:100), BAD (sc-8044; Santa Cruz Biotechnology, C7, 1:100), NOXA (sc-56169; Santa Cruz Biotechnology, 114C307, 1:100) antibodies were used to detect the corresponding proteins in the protein total level and the protein complex assays. To measure BCL2-BAX complex level, anti-BAX (5023S; Cell Signaling Technology, D2E11, 1:100) and anti-BCL2 (BMS1028; Invitrogen, Bcl-2/100, 1:100) antibodies were used to immobilize and detect the BCL2-BAX complex, respectively. Anti-rabbit IgG with Cy3 conjugation (111-165-046; Jackson ImmunoResearch, 1:1,000) and anti-mouse IgG with Cy3 conjugation (715-165-151; Jackson ImmunoResearch, 1:1,000) were used to label the detection antibodies for immunoassay. Anti-BCL2 antibody (BMS1028; Invitrogen, Bcl-2/100, 0.5 μg), mouse IgG1 kappa isotype control (554121; BD Biosciences, 0.5 μg), and anti-mouse IgG with PE conjugation (715-116-150; Jackson ImmunoResearch, 1.5 μl) were used to detect the protein levels for quantitative flow cytometry. Anti-BCL2 antibody (sc-7382; Santa Cruz Biotechnology, C2, 1:1,000) and HRP-linked anti-mouse IgG (7076S; Cell Signaling Technology, 1:1,000) were used to detect the protein levels for Western blotting.

Validation

All antibodies are commercially validated and published previously for their corresponding applications for specific species. All antibodies were validated by immunoassay using the SMPC within our lab described in Extended Data Fig. 1, Extended Data Fig. 3 and Extended Data Fig. 5. All informations about antibodies can be found in the manufacturer's guidelines.

Eukaryotic cell lines

Policy information about cell lines and Sex and Gender in Research

Cell line source(s)

HL60, THP-1, U937 and Ramos cells were purchased from Korean Cell Line Bank. HEK293T and SU-DHL8 cells were purchased from ATCC. NB4 cells were purchased from DSMZ. PC9 cells were provided by Y. Hayata (Kyushu University Faculty of Medicine, Japan). PC9 cells are not currently commercially available.

Authentication

The cell line was authenticated at source, and regularly validated by morphological analysis.

Mycoplasma contamination

The cell line was confirmed to be mycoplasma-negative.

Commonly misidentified lines
(See ICLAC register)

No commonly misidentified cell lines were used.

Flow Cytometry

Plots

Confirm that:

☒ The axis labels state the marker and fluorochrome used (e.g. CD4-FITC).

☒ The axis scales are clearly visible. Include numbers along axes only for bottom left plot of group (a 'group' is an analysis of identical markers).

☒ All plots are contour plots with outliers or pseudocolor plots.

☒ A numerical value for number of cells or percentage (with statistics) is provided.

Methodology

Sample preparation

Viability analysis: All cells were rinsed with cold DPBS and collected by centrifugation. After the supernatants were discarded, the cell pellets were resuspended with cold AnnexinV-binding buffer (Biolegend). After resuspension, AnnexinV-FITC solution (Biolegend) and PI solution (Biolegend) were added into the cell suspensions. The cell suspensions were incubated for 15 minutes at room temperature avoiding exposure to light. After that, 400 µl of cold AnnexinV-binding buffer was added to avoid overstaining.

Quantitative flow cytometry: All cells were rinsed with warm DPBS and collected by centrifugation. After the supernatants were discarded, the cell pellets were resuspended with staining wash buffer. After resuspension, the cells were permeabilized by PW buffer (BD Bioscience) and stained by adding anti-BCL2 antibody or an isotype control antibody. The cell suspensions were incubated for 30 minutes at room temperature avoiding exposure to light. After that, the cell suspensions were rinsed with PW buffer and stained by adding PE-conjugated anti-Mouse IgG with 30 minutes incubation. The stained cells were rinsed with PW buffer and fixed by Fix buffer (BD Bioscience).

Instrument

Flow cytometry was performed on SH800S (Sony).

Software

Analyses were performed using dedicated software for SH800S (v2.1.5, Sony).

Cell population abundance

The purity of the analysed primary samples was confirmed by flow cytometry.

Gating strategy

Viability analysis: The proportions of negatively stained cells for both AnnexinV and PI from each primary sample were calculated and converted to viability (%) (see Extended Data Fig. 4a).

Quantitative flow cytometry: Quantum R-PE MESF beads (Bangs Laboratories) were used to produce calibration curves. These curves were subsequently employed, following the manufacturer's instructions, to quantify the levels of BCL2 in the samples (Smith, 2019).

☒ Tick this box to confirm that a figure exemplifying the gating strategy is provided in the Supplementary Information.
